# Strigolactone: An Emerging Growth Regulator for Developing Resilience in Plants

**DOI:** 10.3390/plants11192604

**Published:** 2022-10-03

**Authors:** Ameena Fatima Alvi, Zebus Sehar, Mehar Fatma, Asim Masood, Nafees A. Khan

**Affiliations:** Plant Physiology and Biochemistry Laboratory, Department of Botany, Aligarh Muslim University, Aligarh 202002, India

**Keywords:** abiotic stress, phytohormones, strigolactones, sustainable agriculture

## Abstract

Improving plant resilience to changing environmental conditions is the primary focus of today’s scientific research globally. It is essential to find various strategies for the better survival of plants with higher resistance potential to climate change. Strigolactones (SLs) are multifunctional β-carotene derivative molecules that determine a range of plant growth and development aspects, such as root architecture, shoot branching, chlorophyll synthesis, and senescence. SLs facilitate strong defense responses against drought, salinity, heavy metal, nutrient starvation, and heat stress. The SLs trigger other hormonal-responsive pathways and determine plant resilience against stressful environments. This review focuses on the mechanisms regulated by SLs and interaction with other plant hormones to regulate plant developmental processes and SLs’ influence on the mitigation of plant damage under abiotic stresses. A better understanding of the signaling and perception of SLs may lead to the path for the sustainability of plants in the changing environmental scenario. The SLs may be considered as an opening door toward sustainable agriculture.

## 1. Introduction

Climate change is a significant threat and will worsen in the future. The consequences of climate change include flooding, drought, higher temperature, irregular rainfall patterns, and others [1,2]. Humans are mobile and therefore easily adaptable to the environment through avoidance; however, sessile plants rely more on resistance than avoidance responses. The global food system has an ample environmental footprint, and agriculture is the most significant sector with the most influential after-effects of ecological imbalance. Researchers are getting too strategic in mitigating crop and nutrient loss. Feeding the growing population and protecting the environment are the ways toward sustainable development. Therefore, the human race is busy finding alternative resources and hidden pathways to reduce the damage and contribute to sustainable development. Plant research these days mainly focuses on strengthening plant immunity against climate change. For this purpose, work is focused on exploring hormonal signaling pathways, gene manipulation, and other proteomic strategies. Abiotic stress also causes alterations in plant morphology and physiology related to plant hormone systems [3]. Strigolactones (SLs) are one of the emerging hormones with much scope in plant resilience. Initially reported as a germination stimulant in parasitic plants, SLs are now in demand for improving plant growth and development [4]. As per the studies, SLs govern the overall plant architecture. SLs influence shoot branching and root structure, modify the phenotypic output of PIN-FORMED (PIN) auxin transporters by inhibiting the formation of auxin-conducting channels after wounding or from artificial auxin sources [5], and monitor secondary growth [6]. SLs also mediate plant response to nutrient deficiency of nitrogen (N) and phosphorous (P) [7]. SLs’ role in improving mycorrhizal colonization in plants makes them distinctive and captivates workers to unravel the complete mechanism of action in living organisms. There are instances where SLs have played an influential role in mitigating plant impairment when exposed to abiotic or biotic stress. This review will focus on pointing out the SL’s role in curbing the extreme environmental condition without much loss of productivity and elucidating the pathways involved in this regulation.

## 2. Strigolactone: History and Background

Strigol is the first characterized SL as a germination stimulant for the root parasitic plant *Striga lutea* [4]. Since then, SLs are mainly known for host–parasitic plant interaction. SLs are βcarotene-derived molecules synthesized in terrestrial plants. In root parasitic plants such as *Striga, Phelipanche*, and *Orobanche* spp. SLs act as germination stimulants released from the host and some non-host plants [8,9]. SLs also enhance arbuscular mycorrhizal fungi (AMF) colonization and act as hyphal branching factors for fungi. This in turn helps in nutrient uptake by fungi in symbiotic association with a plant. This mechanism, in many ways, improves plant nutrient content, growth, and development [10,11]. Moreover, SLs have a pivotal role in certain non-mycotrophic plants by improving stress responses and general plant growth [12].

**Figure 1:**
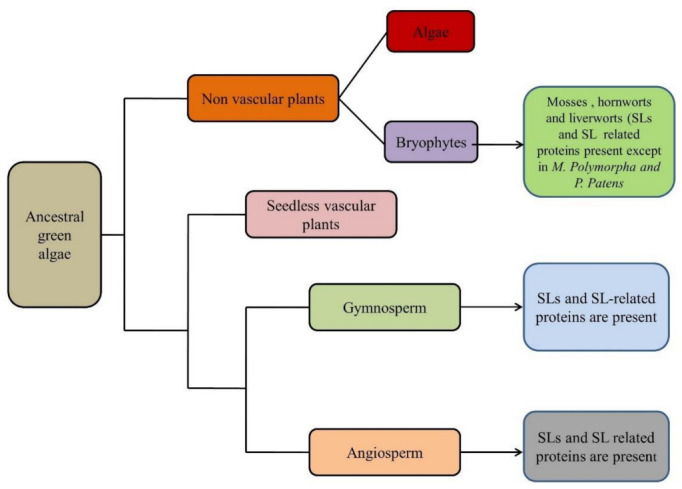
Evolutionary scheme showing common ancestry (presence of either SLs (strigolactones) or SLs-related proteins in land plants).

SLs play key roles in several developmental pathways. The analysis demonstrated that SLs synthesis is reported in liverworts, mosses, lycophytes, gymnosperms, and angiosperms with the core set of SL biosynthesis enzymes [13]. On the contrary, core synthesis enzymes are absent in some species such as *Marchantia polymorpha*, *Marchantia paleacea*, and *Physcomitrella patens* but can synthesize SL through a non-canonical pathway [14,15,16]. For algae, it is suggested that predecessors of *Suppressor of MAX2 1-Like* (SMXL) are present in charophytes, but specific SMXL are absent [17,18] (Figure 1). So far, it is hypothesized that SLs are produced only in land plants, with consistent evolution of true SL biosynthesis enzymes at the base of land plants.

## 3. Strigolactone Biosynthesis and Signal Perception

SLs are tricyclic lactone structures (containing rings as ABC), with different carbon A-ring sizes and substitution patterns on AB-rings. An enol ether bridge connects the core to an α, β-unsaturated furanone moiety (the D-ring). So far, more than 30 SLs have been identified as canonical and non-canonical SLs based on the complete ABC-ring system’s presence or absence [19,20]. Twenty naturally occurring SLs have been identified and characterized so far in root exudates of various land plants. They are separated into two major groups–(a) strigol and orobanchol (ORO) (canonical SLs)–based on the stereochemistry of the B–C-ring junction, with both having a conserved R-configuration at the C-2′ position. The region that connects the D-ring to the core is responsible for the various SLs bioactivities, which can differ according to SL type [21]. In contrast, non-canonical SLs lack typical ABC-rings but possess an enol-ether bridge and D-ring moieties such as methyl carlactonoate (MeCLA) and avenaol [22,23,24].

A series of recessive mutants with increased shoot branching responses helped to understand SLs biosynthesis. The mutants include Arabidopsis *more axillarygrowth (max)*, pea *ramosus (rms)*, petunia *decreased apical dominance (dad)*, and rice *dwarf/high tillering dwarf (d/htd)*. At first, Arabidopsis *max3* and *max4*, the pea *rms5* and *rms1,* and the rice *d17* and *d10* mutants, defective in *CAROTENOID CLEAVAGE DIOXYGENASE 7 (CCD7)* and *CCD8*, respectively, were identified to be SL-deficient mutants [25,26]. Later on, the role of DWARF27 (*D27)* in rice and *AtD27* in Arabidopsis was also reported in SLs biosynthesis [27,28].

**Figure 2:**
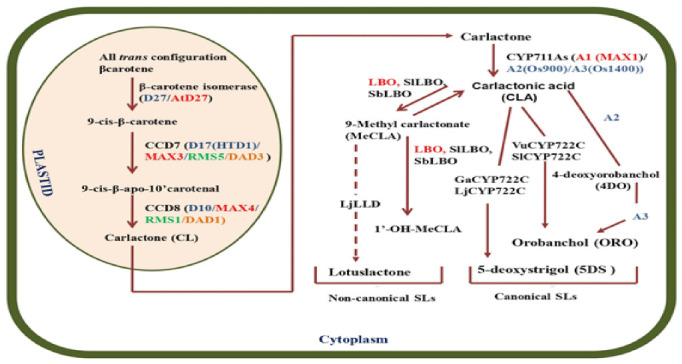
**Strigolactone biosynthesis in a plant cell.** Proposed SL biosynthesis pathway. D27, CCD7, and CCD8 are plastid localized enzymes that form CL from all-*trans* β-carotene. CL is then oxidized by the CYP711A family to yield CLA. GaCYP722C and LjCYP722C are involved in the production of strigol-type canonical SL, 5DS. For non-canonical SLs, MeCLA was shown to be synthesized from CLA in Arabidopsis. It has recently been demonstrated that Arabidopsis, tomato, and sorghum LBOs convert MeCLA into 1-OH-MeCLA and CLA. Moreover, LjLLD, encoding a novel 2OGD, was shown to be involved in the biosynthesis of lotuslactone (non-canonical SL). Enzymes of rice, Arabidopsis, pea, petunia, and other plants are shown in blue, red, green, and orange, respectively. Solid arrows indicate the confirmed pathways, whereas dashed arrows indicate the pathways which are not fully established. LBO (LATERAL BRANCHING OXIDOREDUCTASE) Vu, *Vigna unguiculata* (cowpea); Sl, *Solanum lycopersicum* (tomato); Ga, *Gossypium arboreum* (cotton); Lj, *Lotus japonicus*; Sb, *Sorghum bicolor*.

SLs biosynthesis occurs in plastids with *trans* β-carotene and carlactone (CL) as the ultimate precursor for other SLs [29,30] (Figure 2). The specific enzymes such as carotenoid isomerase, (D27), which has been characterized so far in rice and *Arabidopsis (At27)* [27,28], can convert all-*trans*-β-carotene into 9-*cis*-β-carotene [31].

9-*cis*-β-carotene then serves as a substrate for carotenoid cleavage dioxygenase, CCD7, forming 9 cis β-apo-10′-carlactone, which directly precedes CCD8 in the strigolactone pathway [31]. In rice, the partial loss of function of SL biosynthesis genes (*High TILLERING AND DWARF 1/DWARF 17*) increases tiller number and grain yield. These genes are essential in determining plant architecture [32]. Through a series of oxygenation steps in the presence of cytochrome P450 (MAX1), carlactone, a butenolide ring-like structure is formed, followed by carlactonic acid (CLA) [33], which eventually gives rise to other types of SLs and SL-like compounds. The CYP711As subfamily of cytochrome P450 oxygenases functions in converting CL into both canonical and non-canonical SLs in vascular plants. CYP711A involvement in SLs biosynthesis was first implicated in Arabidopsis. As per the report, the Arabidopsis *max1* mutant, defective in CYP711A1, exhibited the hyper-branching phenotype as *max3* and *max4* [34]. MAX 1 functions downstream of MAX3 and MAX4 as suggested by grafting experiments [35]. Moreover, the drastic increase in the endogenous CL level in *max1* mutant suggested that CL is a substrate for MAX1 [36]. In rice, five homologs of MAX1 with diversified functions have been reported. One of these, Os01g0700900, functions as a carlactone oxidase that converts carlactone to 4-deoxyorobanchol, the precursor for orobanchol-type SLs [29]. A second MAX1 homolog, Os01g0701400, catalyzes the conversion of 4-deoxyorobanchol to orobanchol [29].

According to recent studies, for canonical SLs CYP722C subfamily plays a role in synthesizing both strigol and ORO [37,38,39]. Moreover, the study of Wakabayashi et al. [37] demonstrated the conversion of CLA to ORO in the presence of VuCYP722C and SlCYP722C via18-hydroxy-CLA in cowpea (*Vigna unguiculata*) and tomato (*Solanum lycopersicum*), respectively. The conversion of CLA to ORO by CYP722Cs needs factor(s) controlling the stereospecificity; stereochemistry of the C ring can be determined by LOW GERMINATION STIMULANT1, a sulfotransferase with unknown function in sorghum (*Sorghum bicolor*) [40]. GaCYP722C was shown to catalyze the reaction from CLA to 5-deoxystrigol (5DS) in cotton (*Gossypium arboreum*) [38]. Moreover, in *Lotus japonicus,* LjCYP722C was reported to function in 5DS biosynthesis downstream of CYP711A9/LjMAX1, which produces 18-hydroxy-CLA via CLA [39,41].

For non-canonical SLs, methyl carlactonoate (MeCLA) is the key intermediate and is reported to be produced from CLA [33]. The metabolic action of LATERAL BRANCHING OXIDOREDUCTASE (LBO), a 2-oxoglutarate-dependent dioxygenase (2OGD) in Arabidopsis converts MeCLA to such a product which inhibited shoot branching compared to the *lbo* mutant but showed a weaker hyper-branching phenotype compared with the *max4* mutant [42]. According to Wakabayashi et al. [43], the SABATH methyltransferase in the clade to which At4g36470 belongs may be involved in the carboxymethylation of CLA and the biosynthesis of these non-canonical SLs in Arabidopsis. The identified At4g36470 revealed high substrate specificity for (11R)-CLA, suggesting the enzyme and its orthologs in other plant species may be involved in non-canonical SL biosynthesis. Recently, hydroxymethyl-carlactonoate (1’-OH-MeCLA) has been identified as an unstable LBO product and readily converted to CLA enzymatically or non-enzymatically [44]. The LBO homologs have shown similar reactions in maize, sorghum, and tomato [42]. In lotus, MeCLA or 18-hydroxy-CLA was converted to lotuslactone via LOTUSLACTONE-DEFECTIVE (LLD) because the *LLD-defective mutant* could not produce lotuslactone [39,41].

**Figure 3:**
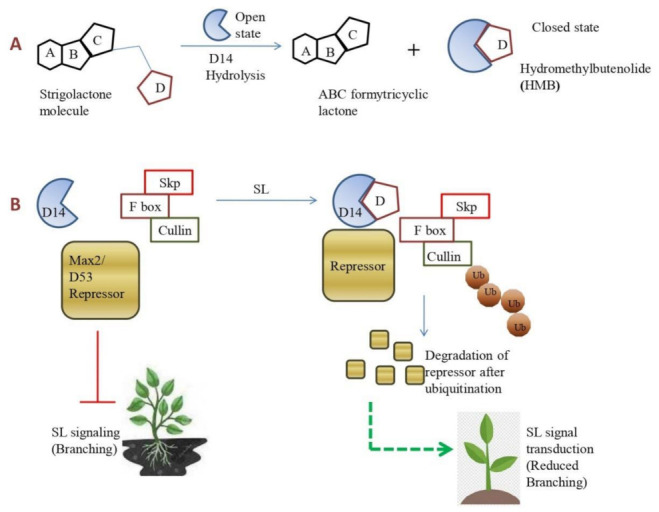
Signal transduction pathway of SLs and protein interaction. (**A**) Degradation of D14 into ABC-FTL and HMB. The figure also shows the open (inactive) and closed (active) conformation of D14 with the “D” ring. (**B**) Interaction of D14 (DWARF 14) with F-box protein D3 and a repressor D53 mediating the signaling pathway. In the absence of SLs, D53 arrests SLs transduction, however, SLs repressor is degraded through controlled ubiquitination followed by SLs release and successful transduction. SLs (Strigolactones); D14 (DWARF 14); ABC-FTL (ABC formlytricyclic lactone); HMB (Hydroxymethylbutenolide); D3 (DWARF 3); D53 (DWARF 53).

Similar to biosynthesis, certain SL-insensitive mutants are characterized, such as *max2* in Arabidopsis, *d3* in rice, and *rms4* in pea for SLs perception. SLs signaling cascade consists of three important components: (a) an α/β fold hydrolase called DWARF 14/DECREASED APICAL DOMINANCE 2 (D14/At D14/DAD2 in rice, Arabidopsis, and petunia, respectively) [45]. DAD2 catalyzes the hydrolysis of the synthetic SL analogue GR24 (b), an F-box leucine-rich protein called MAX2/D3 [34,46], and (c) a repressor protein called D53 belonging to the SMXL protein family [47]. The SL receptor protein D14 is activated after ligand binding, leading to its interaction with other molecules to form a signaling complex; hormonal signal transduction is followed by subsequent hydrolysis of the bound SL, deactivating the hormone [48]. α/β-fold hydrolase, D14 (D14/AtD14/DAD2), and F-box protein (MAX2/D3/RMS4) act as a recognition subunits in an SKP1-CUL1-F-box-protein (SCF)-type ubiquitin ligase complex [49]. This complex further activates the 26S proteasome and degrades transcription repressors, such as the *Suppressor of MAX2 1-Like* (*SMXLs* *6*,*7*, and *8* in Arabidopsis [50] and *D53* in rice [51]). The signal transduction process begins with the binding of SL to the “open state” pocket of D14/AtD14, and the α/β-hydrolase receptor. The interaction with SL induces deletion of the ABC ring (ABC formyltricyclic lactone) and enabled the D ring to remain tightly and permanently attached to the D14/AtD14 (hydroxymethylbutenolide (HMB)). This closed state conformation of D14/AtD14 triggers interaction with the D3/MAX2-based SCF complex (SKP1-CUL1-F-box-protein (SCF)-type ubiquitin ligase complex) [52]. SCF complex targets the D53 and D53-like SMXL repressor proteins for proteasomal degradation, followed by activation of SL signal transduction and responses [47,51,53] (Figure 3). SLs in the roots are acropetally relocated from the rhizosphere to shoot through the PDR1 transporter [54]. The hydrolytic degradation of SLs was confirmed to be a common reaction catalyzed by the D14 family proteins [55,56,57].

**Figure 4:**
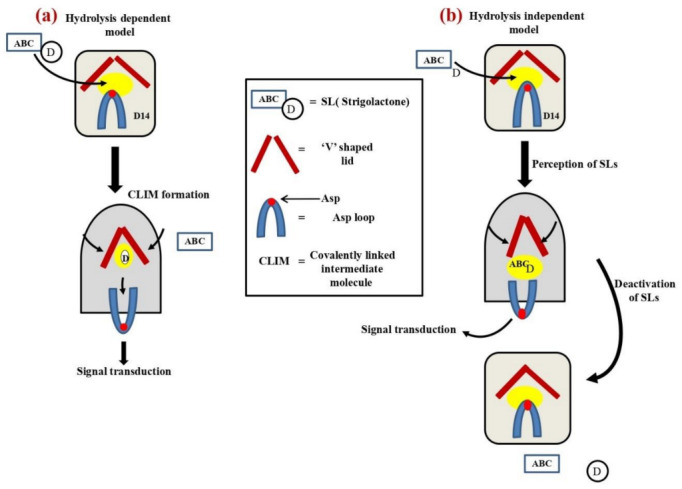
Two different models of SL perception showing the conformational change in D14. The figures are modified from Mashiguchi et al. [30]. (**a**) The hydrolysis intermediate derived from the D-ring part of SLs covalently linked with the receptor, D14, and resulted in a conformational change [52]. (**b**) The intact SL molecules cause a conformational change in D14, and D14 then returns to its catalytically active form [57].

In support of the above view, Yao et al. [52] demonstrated a covalently linked intermediate molecule (CLIM) model for SLs perception (Figure 4a). AtD14, four α-helices form a V-shaped lid structure, and a loop containing the aspartate (Asp) residue of the catalytic triad (serine, Ser; histidine, His; and Asp (Asp loo)) exists between this V-shaped structure; Ser is present at the bottom of the pocket in the open conformation. It was expected that the methyl butenolide part (D-ring) of SL would be the target of the nucleophilic attack by the Ser residue [58]. According to the author, the SL-derived D-ring part is covalently linked to form a bridge between the Ser and His residues which is necessary for the complex formation with D3. However, Carlsson et al. [59] reanalyzed the reported D14–D3–ASK1 complex structural data, and they found that the electron density found in the pocket was too small to accommodate the proposed intermediate molecule [59]. The hydrolysis of D14 was reported to be extremely slow compared to the degradation of the repressor proteins D53/SMXLs [47,51]. Thus, the CLIM model seemed to be inconsistent with this rapid response because this model requires the hydrolysis reaction by D14 to transmit the signal.

Seto et al. [57] proposed the hydrolysis independent model of SL perception (Figure 4b). The catalytically inactive *AtD14^D218A^* mutant was able to complement the *atd14* mutant phenotype in an SL-dependent manner, demonstrating that the hydrolytic degradation of SL by D14 is not necessary for its signal transduction. The presence of the enlarged pocket was observed in the AtD14-D3 protein complex, and the pocket was shown to have enough capacity to accommodate the intact SL molecules. The induction of the D14 active state is triggered by an intact SL molecule, not by the hydrolysis intermediate or products. The intact SL, D14, initially adopts a destabilized conformation due to the disruption of the catalytic triad formation. This form is the active state for SL signaling. In this state, the conformationally altered D14 protein interacts with its signaling partners, D53/SMXLs and D3/MAX2, to transmit the SL signal. The complex D53/SMXLs may bind around the Asp loop region. After the signal transduction, D14 returns to the apo form, which is enzymatically active and deactivates the SL molecules by hydrolytic degradation. The signaling mechanisms of SLs are mediated by a catalytically active α/β-hydrolase (D14) responsible for both the perception and deactivation of bioactive hormone signals. Moreover, yet another model based on the conformations of the D3 F-box protein was proposed by Shabek et al. [60]. Here they showed that the C terminal helix (CTH) of D3 has a more flexible structure, and this part interacts with D14 in an SL-dependent manner. The hydrolysis of D14 is inhibited in the D14-D3 CTH complex. However, it is reactivated in the presence of D53, and the crystal structure of D14-D3CTH induced by SL showed the presence of SL in the active site of D14. This allowed them to conclude that the signal transduction does not require SL hydrolysis and the signaling complex formation process does not require the conformational change of D14, which was different from Seto et al. [57] findings. Burger and Chory [61] reanalyzed the structural data of the AtD14–D3–ASK1 complex reported by Yao et al. [52] and proposed another model in which the D-ring part is covalently attached to only the His residue. It is difficult to integrate all these models and obtain consistent signaling and perception at this point. The SLs perception model is still under discussion and needs further research to understand the mechanism completely.

## 4. Strigolactone Is an Essential Plant Hormone in Regulating Plant Functions

SLs have been popularly known for their germination property in parasitic plants. However, in recent years, several other aspects of plant growth and development have been associated with it. Some of them are listed below.

### 4.1. Root Architecture

A robust root system provides proper anchorage to plants and efficient mineral transport acropetally. Moreover, plants also show some modification and particular root structures depending upon the adaption in parallel with their habitat. Plant hormones, such as auxin, play critical roles in regulating root growth [62]. Similarly, SLs influence various features and plants’ overall root system development. The significant role of SLs has been discovered in primary root formation, lateral and adventitious root development, and root hair elongation. For instance, in Arabidopsis, exogenous application of GR24 (synthetic SL) resulted in longer primary roots. The response was because of increased cell number in transition and meristematic zone [63]. Exogenous application of GR24 suppressed adventitious root formation in Arabidopsis and cut pea stem [64]. Auxin gradient is the chief regulator of root system architecture. For instance, the auxin concentration gradient determines the cell size in transition, meristematic zone, and adventitious root formation [64]. In the presence of auxin, the plant was reported with *CCD8* (SL biosynthesis gene) in root cortical and epidermal cells of transition and elongation zone. Moreover, auxin can promote adventitious rooting even in the absence of SLs, and SLs can suppress adventitious rooting even in the presence of high auxin content. This means both are independently regulated. An auxin response mutant (*axr1*) and SL mutant (*max 3*) double mutant lines tested resulted in no roots similar to *axr1* mutant lines, suggesting an AXR1-dependent pathway of MAX action. Root hair elongation after SLs application in wild line and SL-deficient line were reported, but not in SL response mutant *max 2* proves that this elongation is facilitated via *MAX 2* gene [64]. In contrast, SLs promote crown root elongation in rice by increasing cell numbers [65].

### 4.2. Shoot Branching

SLs play a significant role in affecting shoot branching. The Branching Inhibiting Signal (BIS) and SLs are similar in their function as they are significant players in apical dominance and are carotene derivatives; thus, they could be at least related [66]. D14 is the first receptor in branch signaling. The signaling is further preceded by the degradation of *SMXLs* *6*, *7*, and *8* [51]. Loss of function of the *SMXL* genes causes a reduction in shoot branching and promotes auxin transport and PIN1 accumulation in the shoot. This suggests that SLs mediate shoot branching in coordination with auxin via SMXLs [67]. BRC1 is a repression factor on auxiliary bud, and SMXL is known to release this suppression and promote branching. Target degradation of SMXL by SLs reverses this effect. BRC1 has been reported in pea, Arabidopsis, and rice species acting downstream of the SLs pathway [68,69]. In maize, the BRC1 homolog *TEOSINTE BRANCHED1* (*TB1*) is reported with a similar mode of action and function [70]. The loss of function mutant and use of SL deficient strains supported the above findings and proved that SLs down-regulate shoot branching.

### 4.3. Leaf Senescence and Photosynthesis

Leaf senescence takes place by the sequence of events such as chloroplast degradation and denaturation of RuBisCo and chlorophyll (Chl) a/b binding protein (CAB), then leaf colour changes to yellow [71]. The role of SL in leaf senescence is proved by the application of GR24 in SL deficient (*d27, d17,* and *d10* in *Oryza sativa* as well as *max1, max3,* and *max4* in *Arabidopsis thaliana*) and SL insensitive mutants (*d3* and *d14* of *Oryza sativa*, and *max2* and *atd14* of *Arabidopsis thaliana*) [71,72]. The normal course of senescence was restored regarding leaf colour, Chl content, and electrolyte leakage in SL-deficient *Oryza sativa* mutants, but not SL-insensitive mutants after GR24 application. The delayed senescence and increased senescence in the presence of GR24 were observed in SL-related mutants, indicating the role of SLs in leaf senescence.

GR24 application restored the level of Chl in drought stress. Parallel observations were also observed in wheat under drought stress [73] and rapeseed under salinity stress [74]. The Chl a/b ratio, an indicator of a plant’s ability to use light energy of different wavelengths [75], was reduced in SL-deficient mutants *(d27, d17, d10, and max1, max3, and max4)* [71,72]. The value of electron transport rate through PSII, Y(II), and NPQ was lower than that in wild type, indicating that SLs could modulate the capacity of leaves for capturing light energy by altering the components of photosynthetic pigments.

### 4.4. Hyphal Branching and Nodulation

AMF formed a symbiotic association with most land plants [76,77,78]. Studies supported that SLs help in inducing hyphal growth in AMF, thereby increasing mycorrhizal colonization in plants and consequently improving nutrient absorption and resource transport. Studies on rhizosphere, the mutant of pea and tomato deficient in SLs production, showed reduced AMF hyphae branches compared to wild type plants [79]. However, in the SL-biosynthesis mutant, lower colonization rates were observed in the wild type. The differences were minimized when the plants were inoculated with spores and hyphae or infected roots [79].

SLs play essential roles in regulating nodules under nitrogen deficiency in legumes [80,81]. In pea, SLs and brassinosteroids biosynthesis genes promote nodulation independently of the autoregulation of nodulation (AON) system [80]. Furthermore, a low concentration of GR24 in alfalfa can significantly increase nodulation number [82]. Likewise, in SL-deficient pea and lotus, GR24 helped maintain nodule formation. Additionally, the SL-deficient *rms4* mutant in pea carries the maximum number of nodules than the wild type in comparison to *rms1* plants [80,81,83]. Moreover, soybean with the over-expression of *GmMAX2a* has a higher number of nodules. In contrast, knockdown strains of the same gene have fewer nodules [84]. Therefore, we can say SLs positively control nodulation in legumes and play an essential role in nitrogen acquisition, nutrient access, and yield in legumes.

## 5. Strigolactones and Abiotic Stress

Generally, plants growing in standard conditions in the laboratory have deficient strigolactone levels. This primary hormone level allows some branching for maximal light capture and limits root growth for sufficient nutrient uptake and structural stability. On the other hand, when the plant encounters specific environmental difficulties, such as suboptimal nutrient availability or abiotic stress, strigolactone levels rise to optimize and adapt the plant’s growth strategy to fit the conditions [85,86]. 

The plant needs both morphological and physiological changes to maintain homeostasis. Researchers have documented SLs’ behaviour under unusual conditions. Some of them are mentioned here.

### 5.1. Nutrient Starvation

SLs have been suggested to plant architecture regulators concerning P regulation. Lateral root formation is repressed during sufficient P availability, whereas it is promoted when P is at low levels in the surroundings [87]. The *phosphorus starvation-induced* (*PSI*) genes are associated with plants responding to low P conditions and are used as markers. The expression of the *PSI* gene is not controlled by P but through SLs. SLs increase P mobility and absorption during severe deficiency. P and N deficiencies induce SLs deposition in root tissues, which activates signaling pathways all through nutrient stress. The pathway involves the expression of *D10*, *D17*, and *D27*, while suppressing *D3*, *D14*, and *D53,* as recorded in rice [88]. Further, in the case of *Arabidopsis thaliana,* the increased transcription of *MAX3* and *MAX4* in N deficiency suggests SLs accumulation in root tissues [7]. The establishment of AMF is one of the most remarkable contributions of SLs in P-deficient soil. The intimacy between the host and AMF is necessary for an influential symbiotic association and efficient P absorption. This association increases mycorrhizal colonization and hyphal growth [89]. Low soil P triggers SLs synthesis in *Trifolium pratense* and induction of *CCD7* in *Zea mays* [90,91]. Similarly, in the case of leguminous plants, nutrient uptake is promoted via excessive nodulation, and in non-leguminous plants, P and N deficiencies facilitate SLs exudation and adopt AM association for nutrient absorption [92].

### 5.2. Drought

In drought stress, the chief avoidance responses include stomatal closure, lower transpiration, reactive oxygen species (ROS) scavenging, reduced lipid peroxidation, controlled Chl content, and photosynthetic rate. SLs have been shown to mitigate drought resulting in damage to many plants. Reduced H_2_O_2_, malondialdehyde (MDA), and electrolyte leakage levels and improved water content, photosynthesis, and membrane stability in the drought-exposed wheat and *Vitis vinifera* after SLs application suggest that it triggers ROS scavenging machinery [93,94]. Likewise, in Arabidopsis, *max* mutant strains showed a higher transpiration rate than the wild type. SLs also regulate Chl components and photosynthetic rate under drought stress [95]. SLs can also alter stomatal closure through abscisic acid (ABA) regulation indirectly. Reduced ABA biosynthesis in the rice strain mutant in the *OsD27* gene suggested some correlation. In the *d27* mutant line, reduced SL and ABA synthesis were recorded with higher drought sensitivity [96]. Similarly, the *D14 mutant of Hordeum vulgare* was hypersensitive to drought stress. In the mutant, lower relative water content, disorganized chloroplast structure, impaired photosynthesis, altered stomatal density, slower stomatal closure, and disrupted ABA metabolism were reported, unlike wild type [97]. Moreover, SLs proved beneficial in improving oil content in *Dracocephalum kotschyi* under drought conditions [98].

### 5.3. Salinity

SLs have also been found effective in controlling damage caused by salinity stress. SL-deficient (*max3* and *max4*) and SL-signaling (*max2*) mutants showed hypersensitivity to salinity in *Arabidopsis thaliana* and *Malus domestica* during the germination and vegetative phase. Moreover, applying SLs in salt-exposed rice [99] and *Salvia nemorosa* [100] improved ROS scavenging responses, such as reduced H_2_O_2_ and MDA content and lesser cellular damage. The findings of Ren et al. [101] state that a combination of salt stress, ABA, and H_2_O_2_ stimulates SL-biosynthesis genes, including *CCD7*, *CCD8* (in root) as well as *MAX2* (in the shoot) in AMF-associated *Sesbania cannabina*. In the presence of H_2_O_2_ and SL inhibitor (TIS108, SL biosynthesis inhibitor), ABA-induced SL synthesis was blocked. Thus, we can conclude that ABA is essential for AMF-induced SL synthesis under salt stress. Likewise, in AMF inoculated *Lactuca sativa* roots SLs and ABA biosynthetic genes showed a positive correlation under salinity stress [102].

### 5.4. Heavy Metals

Heavy metal toxicity is one of the significant challenges in the agriculture sector. It retards growth, photosynthetic rate, Chl content, and antioxidant activities and increases plants’ reactive oxygen species (ROS) production. For instance, Cd stress in *Panicum virgatum* causes suppression in growth attributes, photosynthetic rate, Chl content, and antioxidant activities while enhancing levels of ROS. However, SLs have been shown to reverse all these symptoms in *Panicum virgatum* against Cd toxicity [103]. In the case of arsenic (As) toxicity, transcript levels of antioxidant enzymes including *OsCuZnSOD1, OsCuZnSOD2*, *OsAPX1*, *OsAPX2,* and *OsCATA* were noted to be relatively higher in wild type roots than SLs mutant (*d10 and d17)* roots in rice plants. The expression of transporter genes (*OsPT1*, *OsPT2*, *OsPT4,* and *OsPT8*) was seen to be higher in mutant plants than in wild type [104]. Structural similarity between As and P causes competitive inhibition of the P transporter, leading to P deficiency in the plant [105]. As stated earlier in this review, P deficiency triggers SL biosynthesis and signaling. Thus, we can assume that the SLs may be involved in the mitigation of heavy metal stress in plants. However, we need more detailed molecular pathways by which SLs act on heavy metal-stressed plants to reduce their harmful effects. Some important genes associated with nutrient deficiency are *D10, D17, D27,* and *OsMAX1,* which stimulate SL biosynthesis.

### 5.5. Heat Stress

Global temperature rise data are alarming to all the developed and under-developing countries. The hike is showing a devastating effect on crop productivity and longevity. Heat stress can sabotage plants’ physiological and morphological processes within a short period if the temperature goes beyond the threshold. During the early stages of plant development, the impact of heat stress is evident as decreased seed germination potential, poor germination, reduced seedling vigour, and, in extreme cases, complete loss of viability [106,107]. Photosynthetic damage, excessive ROS generation, electrolyte leakage, membrane permeability, and chlorosis are the ways by which heat stress can stun plant growth and productivity. To cope with the extreme temperature conditions, plants adopt several strategies, such as the synthesis of heat shock factors and heat shock proteins [108,109], involvement of hormones [110,111,112], activation of enzymatic and non-enzymatic antioxidant systems [113,114], and the accumulation of osmolytes, such as proline and betaine and glyoxylate. SLs bring noticeable changes in plants such as root and shoot architecture patterning [7], responses to nutrient (N and P) deficiency [7], and leaf senescence [115]. Studies showed that cold and heat stress resulted in higher *CCD7* and *CCD8* gene transcription in tomatoes suggesting that SLs positively regulate tomato heat and cold tolerance responses [116].

To have a clear view of SLs signaling and regulation, gene silencing experiments were performed with *CCD7, CCD8, MAX1,* and *MAX2*. According to the results, silent lines were prone to water loss under dehydration and higher stomatal conductance. The increased stomatal conductance and sensitivity of plants transformed with empty vector (pTRV) pTRV-*MAX2* leaves to dehydration relative to pTRV-*CCD7*, pTRV-*CCD8*, and pTRV-*MAX1* leaves are probably due to the role of MAX2 link not only to the strigolactone pathway but also to the (KARRIKIN INSENSITIVE2) KAI2-dependent signaling [117]. The *ccd7* mutant in tomatoes showed increased lateral branches, reduced plant height, and higher stomatal conductance than the wild type. However, then the roots of these plants were treated with GR24 under heat stress, and heat-induced wilting was alleviated in both plants [116]. Heat stress resulted in a decrease in pigment system (PS) II efficiency, quantum yield, and increased REL was overcome in wild type and *ccd* mutants. Accumulation of HSP70 after GR24 exposure has been found to increase, improving heat tolerance responses [116].

SLs regulation under abiotic stress has been the main focus of current studies. Its role under drought and salinity stress has been extensively studied; however, its modulation under heat stress is still emerging. A thermo-inhibition study on Arabidopsis seed revealed the promotive role of SLs in seed germination [118]. The possible coordination between hormones suggested that SL application reduces the ABA/GA ratio, which alleviates seed thermo-inhibition. SLs act upstream of these essential seed hormones to inhibit ABA synthesis and stimulate gibberellin (GA) accumulation. It is the ABA/GA ratio that SLs regulate to break seed dormancy in both parasitic and non-parasitic plants. SLs may suppress the expression of ABA biosynthesis gene 9-cis-epoxycarotenoid dioxygenase (*NCED9)*, resulting in a lower ABA/GA ratio. SLs application also enhanced the level of cytokinin (CK). In parasitic plant findings, CK and ethylene (ET) can stimulate seed germination in some *Striga* spp. including *S. asiatica* and *S. hermonthica,* even without SLs, which means they work downstream of SLs [119]. In the case of Arabidopsis, CK-induced Et biosynthesis facilitates seed germination under heat stress [118].

Another germination experiment was performed on lupine seeds under normal and heat-stressed conditions, and different physiological responses were recorded after SLs application. SLs effectuate specific changes such as higher germination indices, enhanced proline content, and reduced lipid peroxidation. GR24 also enhanced antioxidant enzyme activity and glyoxalase systems in lupine seedlings. The Chl, a fluorescence transient analysis (JIP-test), indicated that *rac*-GR24 provides strength to the oxygen evolution complex and prevents the inactivation of PSII reaction centres, thus conferring heat stress resistance in lupine seedlings [120].

SLs regulation on root development under different temperature conditions was proved by Hu et al. [121] in tall fescue. The result showed crown root elongation, increase in cell numbers, higher transcript of cell cycle-related genes, and down-regulation of auxin transport-related genes in crown root tips of tall fescue. Regulation of cell cycle and auxin transport are the primary targets of GR24 in these plants. Former studies demonstrated root elongation after SLs application by stimulating cell division in the root meristem zone [63,122]. According to this study, SL played a notable role under heat stress in overcoming damage associated with cell cycle genes such as proliferating cell nuclear antigen (*PCNA), Cyclin D (CycD2),* and *Cyclin dependent kinase (CDKB)*. The expression level of these genes is accelerated after strigolactone exposure under heat stress compared to normal conditions [121].

**Figure 5:**
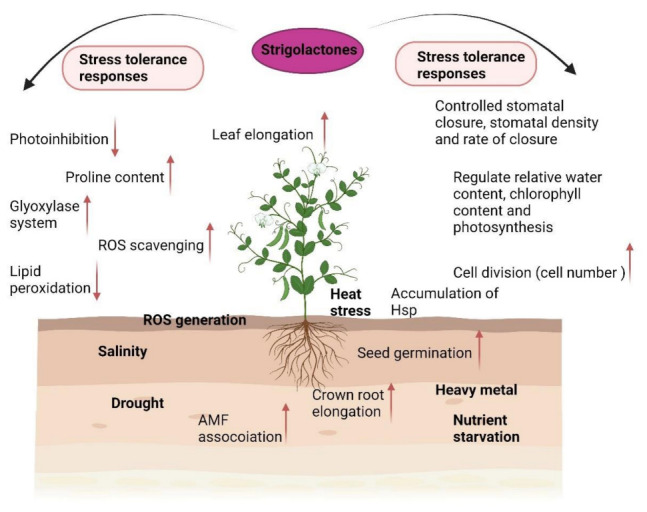
Involvement of strigolactones in plant adaptation to a range of abiotic stresses. ROS (Reactive oxygen species); AMF (Arbasscular Mychorrizal Fungi). Arrows: 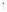 Increase; 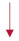 Decrease.

SLs have shown visible effects in determining leaf morphology in the case of both monocots and dicots. Synthetic SLs have shown elongation of internodes [123], mesocotyl [124], hypocotyl [125], and roots [126] through active cell division and proper regulation. SL deficient mutants (*max 1,3,4*) plants are recorded to have reduced petiole and shorter leaf blades than wild type in Arabidopsis [127]. Strigolactone application resulted in increased leaf area under normal conditions; however, its application conferred resistance to heat stress on leaf elongation as explained in the case of root elongation in tall fescue plants [128]. Table 1 provides a summary of the effects of SL on plant functions under abiotic stress. Figure 5 provides the summary of SLs’ response under abiotic stress.

## 6. Interaction of Strigolactones with Other Hormones

### 6.1. Auxin

Auxin regulation in root elongation through SLs has been well documented by workers [63,126]. In tall fescue plants, the combination of SLs and 1-*N*--naphthylphthalamic acid (NPA, auxin transport inhibitor) enhanced root elongation under heat stress. The combined GR24 and naphthaleneacetic acid (NAA) treatment did not enhance root elongation under non-stress or heat stress. These results suggested that SLs could not directly reverse the adverse effects of NAA on crown root elongation in tall fescue but through inhibition of NAA transport. The transcriptional study of the PIN protein family of auxin transporters states suppression of *TIR1, PIN1, PIN2,* and *PIN5* under non-stress and heat stress conditions and positive regulation of auxin on SLs signaling via the *D3* gene, similar is the case with leaf elongation [121,128].

SLs inhibit shoot branching by regulating auxin transport. Auxin transport inhibitor NPA prevents bud out-growth in *max* mutants in Arabidopsis and *dwarf* mutants in rice [27,134]. SLs application reduced basipetal auxin transport and PIN1 accumulation in the plasma membrane in *biosynthesis* mutants but not in *max2* [135]. This proves that SLs dampen the PAT stream in a MAX2-dependent manner [135]. SLs modulated PIN cycling between the plasma membrane and endosome [136]. SLs action was simulated to increase the PIN1 removal rate from the plasma membrane in shoot branching mutants. The study concluded showed that SLs’ action in shoot stimulation depends upon auxin transport status and SLs concentration [137].

### 6.2. Cytokinin and Ethylene

CKs and SLs have generally been found to behave antagonistically in plant systems. Auxin, CK, and SLs have shown a significant role in bud formation. Here auxin and SL inhibit lateral bud growth, whereas CK promotes it. Therefore, tight regulation of the three is needed for standard plant architecture. SLs inhibit bud growth by suppressing CK biosynthesis, as reported in *Zantedeschia aethiopica* and rice. The transcription factors such as *AXR1 and BRC1*, which upregulate SL synthesis and are found in less branched varieties, have been shown to suppress CK synthesis. On the other hand, the concentration of CK biosynthesis enzymes in peas is higher in SL mutant (*rms 1,2*) plants than in normal. This suggests the antagonist mechanism of CK and SL regulation in plants. In Arabidopsis and pea, *BRC1* is suggested to be expressed in axillary buds and act downstream of SL signaling during shoot branching inhibition [68,69,138]. *BRC1* expression is upregulated by SLs application whereas downregulated by CK [69,138].

Parasitic plant seed germination is initiated when they are close to the host. The complex hormonal exchange takes place to break this dormancy. CK and ET can promote this seed germination process in parasitic plants in the absence of SLs [119,139,140], which means they act downstream of SLs; however, CK-induced seed germination is mainly due to enhanced ET biosynthesis [139].

### 6.3. Gibberellins

Both SLs and GAs regulate plant structure and function. According to some workers, GA negatively regulates endogenous SL levels in plants. The crosstalk between SLs and GAs could be linked with *SLENDER1 (SLR1)*, a representative of DELLA proteins that negatively regulate GA signaling because SLR1 might be degraded in an SL-dependent manner, similar to how it occurs in the GA signaling pathway [141]. The GA receptor *GIBBERELLIC ACID INSENSITIVE1 (GID1)* and GA molecule together stimulate the interaction of the GID1 and DELLA proteins and cause its degradation by the 26S proteasome complex. In GA insensitive rice mutant higher level of SL is recorded with semi-dwarf phenotype and increased tillers [142]. According to Ito et al. [143], SL biosynthesis is regulated by GA through GA receptor *GID1* and F-box protein *GID2* dependent manner also, GA treatment reduced the infection of rice plants by the parasitic plant witchweed (*Striga hermonthica*) [143].

Moreover, the SL level was reduced after the application of active GA in the same plant. This indicates that SLs probably regulate shoot branching in cooperation with GAs. In *Arabidopsis thaliana,* promoter regions of SL biosynthesis genes contain fewer motifs recognized by GA-dependent transcription factors. Microarray data analysis has shown that treatment with GA_3_ resulted in varied expression of *Arabidopsis thaliana* SL-biosynthesis genes but in a dose-dependent manner [144]. Final confirmation of the crosstalk between SLs and GAs needs genetic analysis of the hormone and interactions of the SL receptor with single DELLA proteins. SLs application could alleviate thermo-inhibition by decreasing ABA levels by suppressing *NCED9* transcript accumulation, increasing GA accumulation through MAX2, and breaking secondary dormancy in Arabidopsis [118]. SLs reduce the ABA:GA ratio, which amplifies germination activity, and GA is sufficient to counteract thermo inhibition in Arabidopsis seeds but is not sufficient to do so in parasitic plant seeds [118].

### 6.4. Abscisic Acid

The coordinating role of SLs with ABA during heat stress has already been discussed in the review; however, ABA helps overcome other stress. Both SLs and ABA are stress hormones and carotenoid derivatives. Changes in ABA levels also induce transcription of protein-encoding genes, including dehydrins, osmoprotectants, salinity, and drought-related genes that boost plant stress tolerance. The de novo synthesis of ABA has also been reported in stressed leaves and roots [145]. A series of physiological mechanisms are controlled via ABA and SLs under normal and uncontrolled conditions [101]. ABA helps protect mycorrhizal roots from stress, a crucial function for symbiosis development, completion of arbuscular formation, and promotion of sustainable plant root colonization. According to Lopez-Raez [146], a higher level of ABA in mycorrhiza, associated with stressed plants, would help to foster stress tolerance while at the same time enhancing and sustaining AM symbiosis. Under salt stress, SLs and ABA are essential for regulating and establishing symbiotic relationships among host plants and AMF. As per reports, exogenously applied ABA under stress may increase the accumulation of SLs. ABA is well known for its accumulation in plants under abiotic stress, especially in dehydration events [147]. In tomato leaves, ABA content increased after heat or cold stress in wild type and *ccd7* plants, and the effect was more evident with the application of GR24. According to the study, heat and cold stress selectively regulated the transcript levels of ABA biosynthesis gene *NCED6*, *Lycopersicon esculentum DEHYDRIN 4* (Le4), and *ABA-RESPONSIVE ELEMENT BINDING* (ABF4) in wild type and *ccd7* plants. The transcription level of *Le4* in *ccd7* plants under hot conditions and *ABF4* in *ccd7* plants under cold conditions showed smaller peaks; however, treatment with GR24 increased the transcript levels of all the genes in wild type and *ccd7* plants in both the temperature conditions. SLs also mediate the activity of the antioxidant enzyme system under heat stress. Increased transcript levels of Cu/Zn-superoxide dismutase (SOD), ascorbate peroxidase (APX), glutathione reductase (GR), monodehydroascorbate reductase (MDAR), and dehydroxyascorbate reductase (DHAR) under optimal or stress conditions in both wild and *ccd7* in the presence of SL prove its role in activating ROS scavenging responses [116].

**Figure 6:**
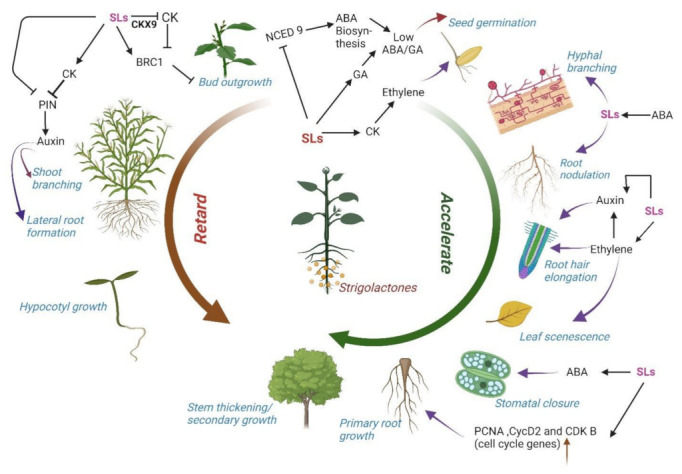
Coordinated role of strigolactones (SLs) with different hormones in regulating plant functions. There are certain phenomena that SLs either retard or accelerate. It needs coordinated crosstalk of different hormones and involvement of various genes to accomplish the outcome. Sometimes SLs may stop the synthesis or signaling of one hormone while promoting the other. In this way, homeostasis is maintained in the plant system in normal and stressed conditions. CKs (Cytokinins); ABA (Abscisic acid); gibberellins (GAs, BRC1 (BRANCH 1); CKX9 (Cytokinin Dehydrogenase); NCED 9 (9); Cyc D (Cyclin D); CDK (Cyclin dependent kinase); PCNA (Proliferating cell nuclear antigen); PIN (PIN FORMED; transporter protein). 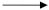 Promotes 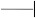 Stops.

Figure 6 shows the possible crosstalk between SLs and other hormones and the relatable outcome after this interaction.

## 7. Conclusions

SLs are essential for proper plant growth and vigour being associated with plant architecture and development, such as seed germination, shoot branching, leaf senescence, root development, and many more. Moreover, they have gained popularity for their significant role in plants’ adaptions to several abiotic stresses, such as drought, salinity, nutrient deficiency, heat, chilling and heavy metals, and in controlling several physiological and molecular processes. Stress may affect SLs biosynthesis, signaling, and crosstalk with other plant hormones. Several hints have recently been reported concerning SLs biosynthesis, but the bioactive form of SLs that regulates various aspects of plant growth and development is still uncertain. There are still many gaps in SLs signaling and perception that must be resolved for sustainable usage in agriculture. Identification of genetic variation and favourable alleles of genes involved in SLs’ diversification and downstream signaling processes would be a valuable asset to future breeding operations. It would aid in fine-tuning to maximize agricultural production. A clear understanding of this will open new doors toward plant resistance and higher yields.

## Figures and Tables

**Table 1:** Effects of strigolactone application at different concentrations on plant functions under abiotic stresses in various plant species.

Plant Specimen	Type of Stress	Mode of Application	Concentration	Effects on Plant	References
*Triticum aestivum*	Drought	Foliar spray	10 μM GR24 (synthetic SL analogue)	Increased: Relative water content, membrane stability index, activities of POD, CAT, and APX Decrease: Electrolyte leakage, MDA	[73]
*Vitis vinifera*	Drought	Foliar spray	1, 3, and 5 μM *rac*-GR24	Increase: Relative water content, Chl content, and rate of photosynthesis, antioxidant capacity, activate transcriptions of *VvHYD1*, *VvHYD2*, *VvCCD7*, *VvCCD8,* and *VvNCED1* Decrease: Electrolyte leakage, stomatal opening, ROS, and MDA content	[93]
*Triticum aestivum*	Drought	Foliar spray	10 μM GR24 (synthetic SL analogue)	Increase: Proline and soluble sugar content, stomatal conductance, photosynthetic rate, osmotic adjustment Decrease: Water potential, transpiration rate, H_2_O_2_	[94]
*Dracocephalum kotschyi*	Drought	Spray	10 μM *rac*-GR24	Increase: Fresh and dry weights, essential oil content, and yield Decrease: Electrolyte leakage, MDA, H_2_O_2_	[98]
*Triticum aestivum*	Salinity	Solution	0.001, 0.01, and 0.1 mg L^−1^ GR24 (synthetic SL analogue)	Increase: CO_2_ assimilation rate, non-photochemical quenching and photochemical quenching, stomatal conductance Decrease: Total leaf area, root length, root fresh and dry weights	[129]
*Brassica napus*	Salinity	Solution	0.18 μM GR24 (synthetic SL analogue)	Increase Leaf Chl content, net photosynthetic rate, stomatal conductance, intercellular CO_2_ concentration and transpiration rate, POD and SOD activities, expression of DEGs Decrease: H_2_O_2_ level, MDA content	[74]
*Oryza sativa*	Salt	Hoagland nutrient solution	0.1, 0.2, 1, and 5 μM GR24 (synthetic SL analogue)	Increase: Plant height, root length, SOD and POD activities, intercellular CO_2_ concentration, net photosynthetic rate Decrease Lateral buds outgrowth, MDA, ROS	[86]
*Salvia nemorosa*	Salinity	Spray	0.1, 0.2, 0.3, and 0.4 μM GR24	Increase: Net photosynthetic rate, stomatal conductance, intracellular CO_2_ and gas-exchange, essential oil yield, Chl content Decrease: MDA, electrolyte leakage, H_2_O_2_, activities of SOD, POD, CAT, and glutathione Reductase	[100]
*Malus domestica* and *Arabidopsis thaliana*	Salt, drought, and low temperature	MS medium	5, 10, and 20 μΜ GR24 (synthetic SL analogue)	Increase: *MdD14* degradation Decrease: Hypocotyl elongation, shoot branching, MdD14-His protein	[130]
*Helianthus annuus*	Salinity	MS medium	0.001, 0.01, and 0.1 mg L^−1^ GR24 (synthetic SL analogue)	Increase: Activities of CAT and SOD, callus biomass, glycine betaine and protein content Decrease: MDA, Na^+^ content, H_2_O_2_	[131]
*Lotus japonicas*	*Osmotic and phosphorous*	Solution	5.0 μM GR24 (synthetic SL analogue)	Increase: *LjPDR1-295a* and *LjPDR1-345* expression Decrease: ABA level, transcript level of ABA biosynthetic gene *LjNCED2,* and ABA catabolic gene *LjAAO3*	[132]
*Oryza sativa*	N	Nutrient solution	2 μM GR24 (synthetic SL analogue)	Increase: *FC1* expression in tiller buds, *OsIPT* transcript level, expression of *OsCKX* Decrease: Tiller bud outgrowth, auxin transport capacity, IAA level, expression of *OsPIN9*	[133]
*Festuca arundinacea*	*Heat*	Foliar spray	0.01 μM GR24 (synthetic SL analogue)	Increase: Cell cycle-related genes, cell number, crown root elongation, expressions of *D3* and *D14* Decrease: Auxin transport related genes (*TIR1*, *PIN1*, *PIN2,* and *PIN5*)	[121]
*Festuca arundinacea*	Heat	Foliar spray	0.01 μM GR24 (synthetic SL analogue)	Increase: leaf elongation, Cell cycle-related genes, cell number expressions of *D3* and *D14* Decrease: Auxin transport-related genes (*TIR1*, *PIN1*, *PIN2,* and *PIN5*)	[128]
*Arabidopsis thaliana*	Heat	Solution	20, 0.1 µM GR24 (synthetic SL analogue)	Increase: Seed germination, P level, GA, and CK accumulation Decrease: ABA/GA ratio ABA levels, secondary dormancy	[118]
*Solanum lycopersicum*	Heat and cold	Solution	1, 3 and 9 µM GR24 (synthetic SL analogue)	Increase: Hsp70, ABA synthesis, transcription of *CBF1*, *CBF3*, SOD, APX, GR, MDAR, and DHAR activity Decrease: heat sensitivity, REL level, MDA content, H_2_O_2_ content	[116]
*Lupinus angustifolius*	Heat stress	Petri plate treatment	3 µM *rac*-GR24	Increase: seed resilience to high temperature, SOD activity, proline content, *glyoxalase I and II activity* PI_abs_, ROS scavenging mechanism Decrease: peroxidase activity, lipid peroxidation, ABS/RC ratio	[120]

## Data Availability

Not applicable.
